# Effect of Surface Modification for Carbon Cathode Materials on Charge–Discharge Performance of Li-Air Batteries

**DOI:** 10.3390/ma15093270

**Published:** 2022-05-02

**Authors:** Kaito Fukushima, So Yoon Lee, Kenichi Tanaka, Kodai Sasaki, Takahiro Ishizaki

**Affiliations:** 1 Materials Science and Engineering, Graduate School of Engineering and Science, Shibaura Institute of Technology, 3-7-5 Toyosu, Koto-ku, Tokyo 135-8548, Japan; mb21045@shibaura-it.ac.jp (K.F.); mb21035@shibaura-it.ac.jp (K.T.); mb22019@shibaura-it.ac.jp (K.S.); 2 Department of Materials Science and Engineering, College of Engineering, Shibaura Institute of Technology, 3-7-5 Toyosu, Koto-ku, Tokyo 135-8548, Japan; soyoon@shibaura-it.ac.jp

**Keywords:** carbon, surface modification, Li-air battery, charge–discharge performance

## Abstract

Li-air batteries have attracted considerable attention as rechargeable secondary batteries with a high theoretical energy density of 11,400 kWh/g. However, the commercial application of Li-air batteries is hindered by issues such as low energy efficiency and a short lifetime (cycle numbers). To overcome these issues, it is important to select appropriate cathode materials that facilitate high battery performance. Carbon materials are expected to be ideal materials for cathodes due to their high electrical conductivity and porosity. The physicochemical properties of carbon materials are known to affect the performance of Li-air batteries because the redox reaction of oxygen, which is an important reaction for determining the performance of Li-air batteries, occurs on the carbon materials. In this study, we evaluated the effect of the surface modification of carbon cathode materials on the charge–discharge performance of Li-air batteries using commercial Ketjenblack (KB) and KB subjected to vacuum ultraviolet (VUV) irradiation as cathodes. The surface wettability of KB changed from hydrophobic to hydrophilic as a result of the VUV irradiation. The ratio of COOH and OH groups on the KB surface increased after VUV irradiation. Raman spectra demonstrated that no structural change in the KB before and after VUV irradiation was observed. The charge and discharge capacities of a Li-air battery using VUV-irradiated KB as the cathode decreased compared to original KB, whereas the cycling performance of the Li-air battery improved considerably. The sizes and shapes of the discharge products formed on the cathodes changed considerably due to the VUV irradiation. The difference in the cycling performance of the Li-air battery was discussed from the viewpoint of the chemical properties of KB and VUV-irradiated KB.

## 1. Introduction

Li-air batteries have attracted considerable attention as rechargeable secondary batteries due to their high theoretical energy density of 11,400 kWh/g [1]. Particularly, Li-air batteries are expected to replace Li-ion batteries for electric vehicles that require a high energy density. However, the commercial use of Li-air batteries is hindered by issues such as low energy efficiency and a short lifetime (cycle numbers) [2]. The significant issues include a high overpotential difference between charging and discharging, types of products generated from the discharging reaction, and low efficiency of the formation and decomposition of such products [2]. To clarify these issues, it is necessary to understand the role of the materials used for electrodes, the electrolytes in the batteries, and other factors [2]. The cathode material is an important part of a cell because it hosts the charging and discharging reactions, which have a significant effect on the performance of Li-air batteries. Therefore, it is important to select a cathode material that facilitates excellent battery performance.

Carbon materials have good electrical conductivity and porosity as well as oxygen-reducing abilities for the discharging reaction; therefore, they are considered as promising candidate materials for cathode materials in Li-air batteries [3,4,5,6]. Among the various properties of carbon materials, it has been reported that specific surface area, porous structure, and crystallinity are important factors affecting the performance of Li-air batteries [7,8,9,10,11]. From the viewpoint of the specific surface area of the cathode material, it is considered that the surface condition of carbon affects the redox reaction of oxygen because an oxygen reduction reaction occurs during discharging and an oxygen evolution reaction occurs during charging [12]. Therefore, it has been reported that there is a relationship between the functional groups on the surface, which affects the condition of the carbon surface and, subsequently, the performance of Li-air batteries [10,13,14]. However, these reports have discussed the issues combined with (1) the change in the functional groups on the surface of carbon materials (chemical change) and (2) the change in the crystallinity and morphology of carbon materials (physical change). Therefore, it was found that only the combined effect of the carbon material (physicochemical effect) considerably affected the battery performance. Consequently, the effect of functional groups on the performance of Li-air batteries has not yet been clarified.

In this study, we selected commercial Ketjenblack (KB) as a representative carbon material for the cathode material and modified the surface functional group to investigate the effect of surface modification on the charge–discharge performance of Li-air batteries. In particular, we investigated the effect of the surface condition of KB on the performance of Li-air batteries.

## 2. Materials and Methods

### 2.1. Preparation of Cathode Materials

A dispersion medium was prepared by 1-methyl-2-pyrrolidone (NMP) containing 1 wt.% polyvinylidene fluoride (PVDF) by stirring for 24 h at room temperature. The dispersion medium was controlled at a weight ratio of commercial Ketjenblack (KB: EC600JD) and PVDF of 1:9. After this step, the carbon slurry was prepared by adding NMP to this medium, and ultrasonication was performed for 30 min to obtain a homogeneous carbon slurry. The obtained carbon slurry was dropped to carbon paper (TORAY Co., TGP-H-060) and dried at 120 °C for over 12 h using a vacuum drying oven to remove any residual solvent. This cathode material is denoted as KB in this study. 

### 2.2. Surface Modification of the Cathode Material 

Surface modification of the commercial Ketjenblack was carried out by irradiating a vacuum ultraviolet (VUV) light with a wavelength of 172 nm for 30 min. After that, the cathode materials using KB after VUV irradiation were obtained through the abovementioned procedures. Hereafter, the cathode materials using KB after VUV irradiation are denoted as KB + VUV in this study. 

### 2.3. Evaluation of the Charge–Discharge Performance

Li-air cells were assembled for the charge–discharge tests. KB and KB + VUV were used as the cathode materials. Tetraethylene glycol dimethyl ether (TEGDME) was dried for several days over freshly activated molecular sieves (type 4 Å) before use. An ether-based electrolyte was prepared in an Ar-filled glovebox (H_2_O and O_2_ levels < 1 ppm) by dissolving 1 M lithium bis(trifluoromethanesulfonyl)imide in TEGDME. Swagelok-type cells (MTI, Tokyo, Japan: EQ-STC-LI-AIR) were assembled in an Ar-filled glovebox (Miwa Seisakusho, Ama, Japan) (H_2_O and O_2_ levels < 1 ppm) using lithium metal foil as the anode, a glass-fiber membrane (Toray Co., Kyoto, Japan: Whatman GF/A) immersed in the electrolyte as the separator, and Ni foam as the gas diffusion layer.

The cell was connected to a charging–discharging device (Hokuto Denko, Tokyo, Japan: HJ1005SD8, HJ1001SD8C), and the performance of the Li-air battery was evaluated. To evaluate the battery performance, a charge–discharge test (cutoff voltage 2.0–4.5 V, current value 0.2 A/g) and a cycling performance (cutoff voltage 2.0–5.0 V, current value 0.2 A/g, cutoff capacity 1000 mAh/g) were performed under an oxygen gas flow rate of 50 mL/min. 

For cathode characterization, the cells were first transferred to the Ar-filled glove box and disassembled inside it to extract the cathodes. The cathodes were then rinsed with DME and dried for further characterization.

### 2.4. Characterization of Cathode Materials

To evaluate the changes in the surface conditions before and after VUV irradiation of KB, contact angle measurement (Kyowa interface: DM-501, Saitama, Japan), X-ray photoelectron spectroscopy (XPS; JEOL JPS-9010MC, Tokyo, Japan), and Raman spectroscopy (JEOL, Tokyo, Japan spectroscope: NRS-5100) were performed. For contact angle measurement, 10 µL of the ultra-pure water with a resistance of 18.2 MΩ was used. The MgKα line was used as the radiation source for XPS, and the measurements were performed under conditions of 10 kV and 25 mA; the C 1s spectrum was used for the charge-up correction of the obtained spectra. An excitation laser with a wavelength of 532.1 nm was used for Raman spectroscopy. 

To analyze the products generated on the cathode surface after the charge–discharge test, the cells after the discharge tests were first transferred to the Ar-filled glove box and disassembled inside it to extract the cathodes, and the cathode material was rinsed with dehydrated dimethoxyethane (DME). After drying, the cathode material was introduced into a general-purpose atmosphere separator (Rigaku, Tokyo, Japan) in a glove box and sealed to prevent contact with the atmosphere. Under this condition, X-ray diffraction (XRD; Rigaku: Smart Lab, Tokyo, Japan) analysis was conducted without interacting with the atmosphere. CuKα radiation was used as the radiation source for XRD analysis; the measurements were performed at 40 kV and 30 mA. Additionally, Fourier transform infrared (FT-IR) spectroscopy (Shimadzu: IRTracer-100, Kyoto, Japan) and a field emission scanning electron microscopy (FE-SEM; JEOL, JSM-7610F) were conducted to evaluate the products formed on the cathode surfaces. FT-IR spectroscopy was conducted from 400 to 1600 cm^−1^; the resolution was 4 cm^−1^. FE-SEM was carried out at an acceleration voltage of 5.0 kV.

## 3. Results and Discussion

### 3.1. Surface Wettability 

**Figure 1:**
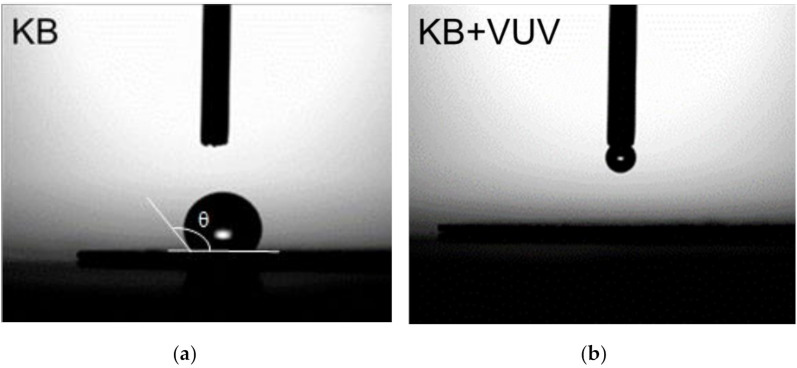
Behavior of a water droplet on (**a**) KB and (**b**) KB + VUV surfaces.

To evaluate the change in the surface condition of KB before and after VUV irradiation, static contact angles of KB and KB + VUV were evaluated. Figure 1 shows the behavior of the water droplets on the cathode materials. As shown in Figure 1a, the static contact angle (θ) of KB was approximately 126°, indicating high hydrophobicity. However, when the water droplets were dropped on the KB + VUV surface, the water droplets immediately wet the surface and spread, indicating that the KB + VUV surface exhibited high hydrophilicity (Figure 1b). Moreover, it can be inferred that a polar functional group having high affinity toward water was introduced onto the carbon surface in the cathode material after VUV irradiation. 

### 3.2. XPS Results

**Figure 2:**
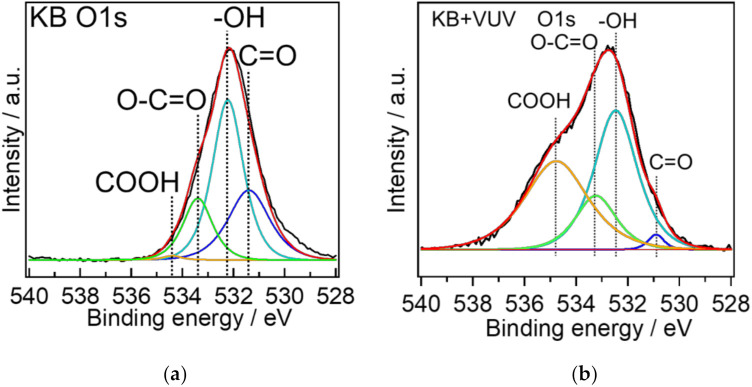
XPS O 1s spectra of (**a**) KB and (**b**) KB + VUV samples. Light green, light blue, blue, orange, and red lines show O-C=O, -OH, C=O, -COOH bonds, and overall fitting, respectively.

**Figure 3:**
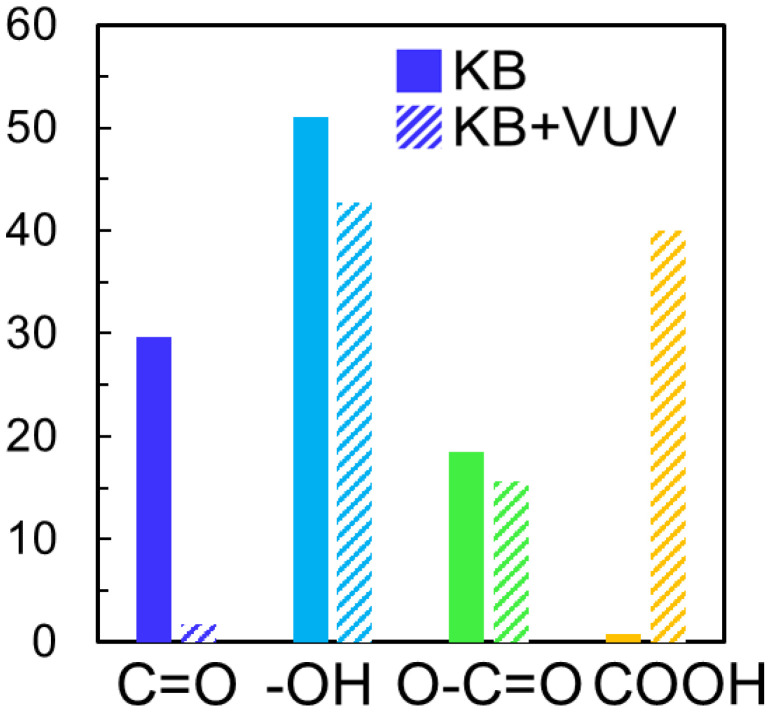
Relative component ratios of chemical bonds for KB and KB + VUV.

The chemical bonding states on the cathode material surfaces before and after VUV irradiation were evaluated by XPS. Figure 2 shows the O 1 s spectra of the cathode materials before and after surface modification. The atomic concentrations of O on the KB and KB + VUV surfaces were estimated to be 15.8 and 19.1%, respectively. This indicates that the atomic O contents increased by the VUV irradiation. One peak was observed in the O1s spectra before and after the VUV irradiation, and the peak was deconvoluted into four components corresponding to C=O, –OH, O–C=O, and COOH bonds at around 531.4, 532.2, 533.4, and 534.5 eV, respectively [15,16]. These bond species are present on the surfaces of both cathode materials; however, the peak area assigned to COOH bonds for KB was very small. Figure 3 shows the ratio of each binding species obtained from the results of the peak separation of the O 1s spectrum. The relative component ratio of COOH bonds for KB + VUV increased to be about 40%. The results of the atomic concentration and the relative component ratio indicate that the existence ratio of the COOH bonds on KB samples increased considerably via the VUV light irradiation. The ratio of the C=O bond decreased on the KB + VUV surface, and the ratio of the functional group derived from COOH increased. However, a slight change in the ratio of other bond species, i.e., -OH and O-C=O bonds, was observed. From these results, it is assumed that COOH was introduced by VUV irradiation; therefore, the KB + VUV surface was hydrophilized.

### 3.3. Raman Spectroscopy

**Figure 4:**
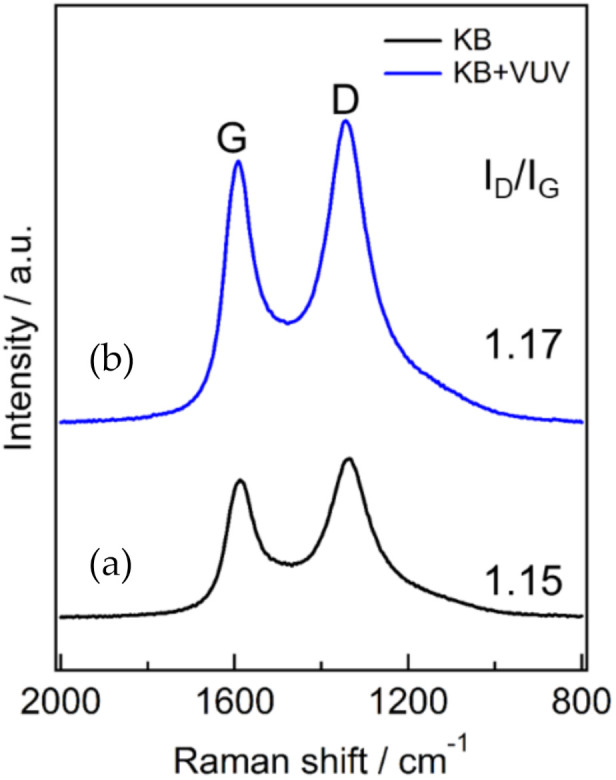
Raman spectra of (a) KB and (b) KB + VUV.

The change in the crystallinity of the KB and KB + VUV was evaluated through Raman spectroscopy. The obtained Raman spectra are shown in Figure 4. The spectra in Figure 4 show two peaks at around 1350 and 1590 cm^−1^ before and after surface modification, respectively. These peaks indicate that the D band at around 1350 cm^−1^ was caused by the defect structure in the crystal plane of carbon and the G band at around 1590 cm^−1^ was due to the graphite structure. The peak intensity ratio (I_D_/I_G_) of the G and D bands is used as an index of the crystallinity of the carbon material [17]; a larger value of this ratio indicates that there are more defects in the material [18]. The I_D_/I_G_ ratios of KB and KB + VUV were almost the same. Therefore, it is considered that the surface modification by VUV irritation had almost little effect on the crystallinity of the carbon material; however, it could change only the functional groups existing on the surface of the cathode material, as shown in Figure 2 and Figure 3.

### 3.4. Charge–Discharge Performances

**Figure 5:**
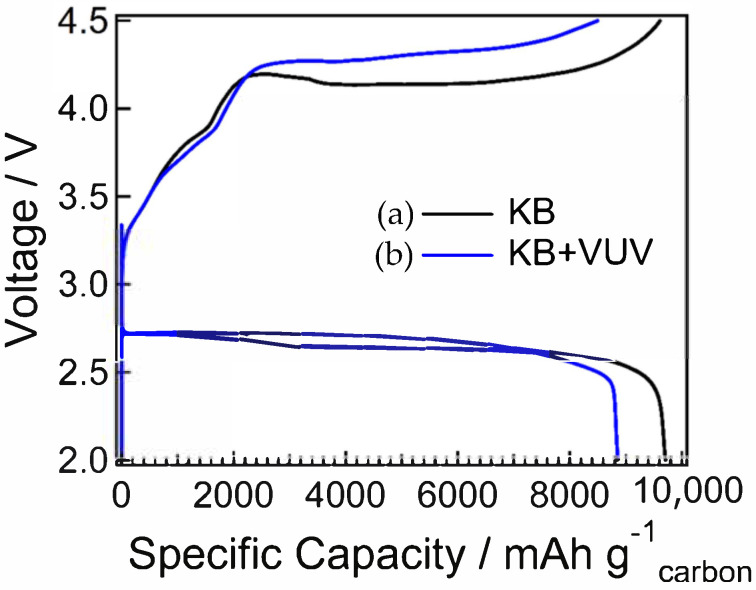
Full discharge and charge curves of Li-air batteries using (a) KB and (b) KB + VUV as cathodes.

**Figure 6:**
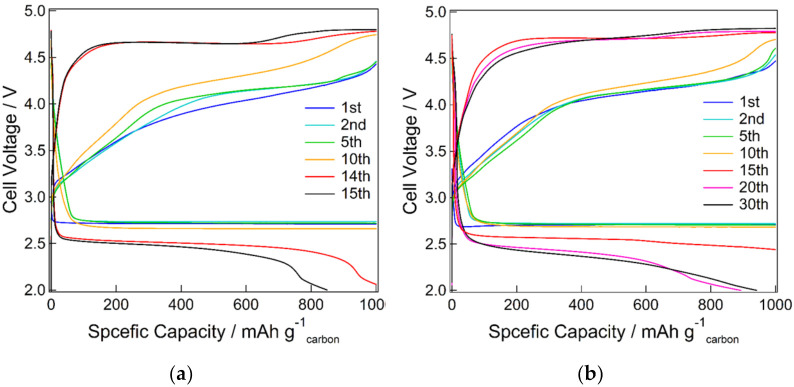
Cycling performance of Li-air batteries using (**a**) KB and (**b**) KB + VUV as cathodes at a limited capacity of 1000 mAh g^-1^.

**Figure 7:**
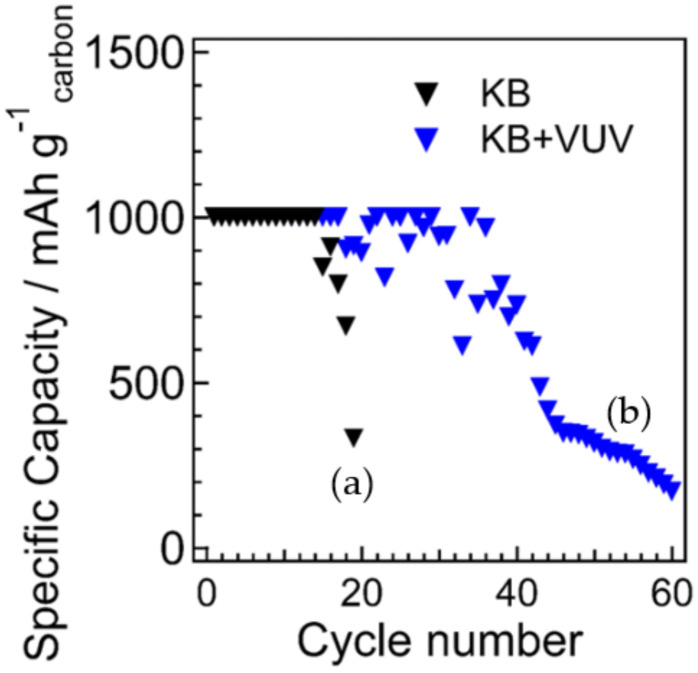
Cycling performance as a function of cycle numbers for Li-air batteries using (a) KB and (b) KB + VUV as cathodes at a limited capacity of 1000 mAh g^−1^.

The full charge–discharge curves are shown in Figure 5. The discharge capacity was estimated to be ~9500 mAh/g for KB and ~8850 mAh/g for KB + VUV, indicating that KB had a slightly larger discharge capacity than KB + VUV. The discharge voltage was ~2.7 V for both samples, and no change was observed before and after surface modification. The charging overpotential was decreased by the surface modification. In addition, the results of the cycling performance are shown in Figure 6 and Figure 7. The discharge capacity of KB began to decrease from the 14th cycle and decreased abruptly after the 15th cycle. After surface modification, the discharge capacity of KB + VUV decreased from the 18th cycle; however, the capacity was maintained until the 37th cycle. This difference in the cycling performance can be attributed to the introduction of hydrophilic functional groups on the carbon surface after surface modification, which has an effect on the charging–discharging reaction in the Li-air battery. Therefore, the change in the surface states of both cathode materials before and after charge–discharge tests were investigated in detail.

### 3.5. Analysis of the Cathode Material Surfaces before and after Charging and Discharging

**Figure 8:**
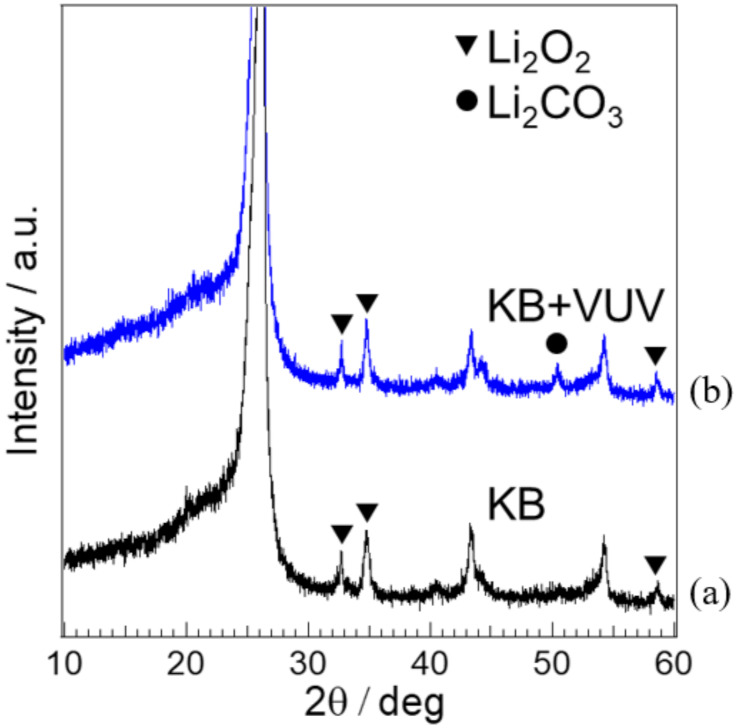
XRD patterns of (a) KB and (b) KB + VUV surfaces after the 1st discharge test.

XRD analysis was performed to examine the discharge products generated on the cathode surface before and after surface modification. Figure 8 shows the XRD patterns of the samples. It has been reported that the crystallinity of lithium peroxide, which is a discharge product, changed depending on the surface states of carbon [10]. Therefore, we unified the discharge time; the discharge capacity was measured at 5.0 mAh to observe the surface states of the cathode materials. On both XRD patterns, three clear peaks attributable to carbon paper were observed at around 2θ = 26°, 43.5°, and 54°. In addition to these peaks, on the XRD patterns of both cathode material surfaces, a few peaks assigned to lithium peroxide were observed at around 2θ = 33°, 35°, and 59°, indicating that the generated discharge product was mainly lithium peroxide. Additionally, in the XRD pattern of KB + VUV, a peak assigned to lithium carbonate was also observed at around 2θ = 50°. This means that the lithium carbonate was also considered to be formed on the cathode sample for KB + VUV. It is considered that the presence of the lithium carbonate formed on the cathode sample for KB + VUV during discharge induced the increase in the overpotential for the charging process of KB + VUV.

**Figure 9:**
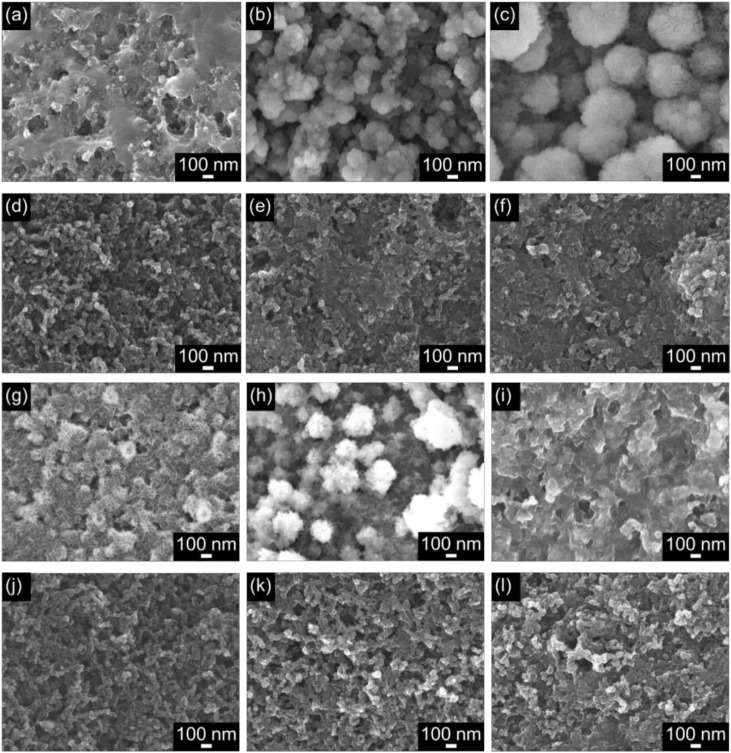
SEM images of (**a**–**f**) KB and (**g**–**l**) KB + VUV surfaces after (**a**) 1st discharge, (**b**) 5th discharge, (**c**) 10th discharge, (**d**) 1st charge, (**e**) 5th charge, (**f**) 10th charge, (**g**) 1st discharge, (**h**) 5th discharge, (**i**) 10th discharge, (**j**) 1st charge, (**k**) 5th charge, and (**l**) 10th charge cycles.

**Figure 10:**
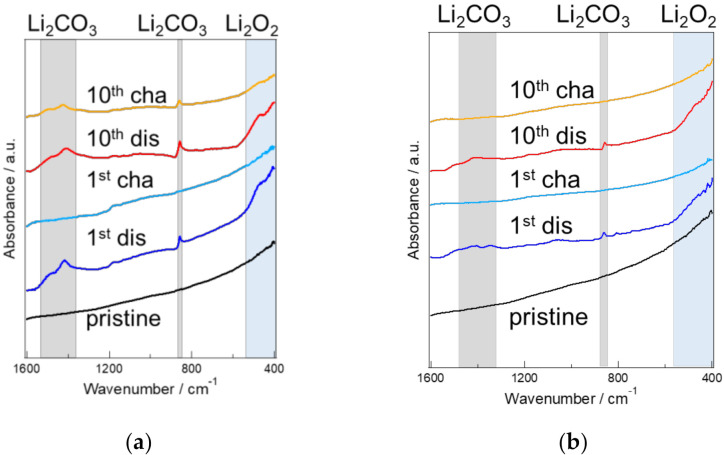
FT-IR spectra of (**a**) KB and (**b**) KB + VUV before and after cycling tests.

The surface states of the cathode materials after charging and discharging were observed using FE-SEM. The SEM images are shown in Figure 9. In the case of KB, thin-film substances were formed on the surface after the 1st discharge cycle (Figure 9a); however, these products were not present on the surface of the cathode material after charging (Figure 9d). Therefore, the discharge products were formed during the 1st discharge cycle, and were decomposed upon further charging. The shape of the products generated on KB during discharge tended to change after the 5th (Figure 9b) and 10th cycles (Figure 9c); large and assembled particles were observed on the cathode material at the 10th discharge cycle. After the 5th charge cycle, the discharge products were not significantly decomposed, and existed on the thin films as flakes. Needle-shaped or flat plate-shaped substances were produced as the discharge products delivered from the 1st discharge cycle of KB + VUV (Figure 9g); the shape of this substance was different from that of the substance formed from KB. After charging, the generated products were rarely observed on the surface of the cathode material, and it was confirmed that the discharge products were decomposed by the charging reaction (Figure 9j); the same decomposition trend was observed for KB and KB + VUV. After the 5th discharge cycle, the discharge products (Figure 9h) were a granular substance (Figure 9b) of the same size as those obtained from KB; however, after the 5th charge cycle, the granular substance was decomposed. Its shape was significantly different from that of KB, indicating that many pores could be confirmed on the surface of the cathode material. The shape of the discharge product generated on the KB + VUV changed after the 10th cycle (Figure 9i); it was identical to that of the thin-film substance after the 1st discharge cycle of KB. After the 10th charge cycle, the thin-film substance was decomposed, and the existence of the pores was confirmed. However, it can be observed that the residual pores were fewer after the 10th charge cycle than those on the surface after the 5th charge cycle. 

Comparing the surfaces of KB with KB + VUV after the 10th cycle charge–discharge tests, the number of pores on the surface was significantly different. Because it has been reported that pores served as a diffusion path for O_2_ and Li ions, it is considered that the number of pores has a significant influence on the charge–discharge characteristics of Li-air batteries.

The chemical bonds of the substances formed on the cathode materials after the charge–discharge tests were analyzed by FT-IR. The FT-IR spectra are shown in Figure 10. A peak derived from lithium peroxide (Li_2_O_2_) as the discharge product was observed at near 550 cm^−2^, and peaks associated with lithium carbonate as the discharge product were present at near 1500, 1450, and 870 cm^−1^ [19,20]. In both KB and KB + VUV, the peaks derived from lithium peroxide (Li_2_O_2_) and lithium carbonate (Li_2_CO_3_) were observed after the 1st discharge cycle; however, the peak intensity derived from Li_2_CO_3_ was lower for KB + VUV than that of KB. Additionally, no peaks derived from Li_2_O_2_ and Li_2_CO_3_ were observed after charging, indicating that the generated product decomposed after charging. Comparing the states after the 10th cycle charge–discharge tests, the peaks derived from Li_2_O_2_ and Li_2_CO_3_ in both KB and KB + VUV after discharging were detected; however, the FT-IR spectra after the 10th charge cycles were different. In the case of KB, a peak corresponding to Li_2_CO_3_ was observed after the 10th charge cycle. However, in the case of KB + VUV, no peak associated with Li_2_CO_3_ was observed after the 10th charge cycle. This indicates that the accumulation of the Li_2_CO_3_-derived products on the KB surface became higher than that of KB + VUV surface with an increase in the cycle numbers of the charging–discharging reaction. Itkis et al. revealed that a superoxide radical (*O*_2_^−^) were produced on carbon materials during the discharging reaction, and epoxy groups and carbonates were generated by the reaction of the radical with the carbon materials, which deaccelerated the charging reaction [21]. Therefore, it is considered that a large amount of Li_2_CO_3_ was generated on the KB surface, which blocked the diffusion path of Li ions and O_2_, resulting in the shortening of the cycling performance. However, in this study, a functional group such as a carboxyl group was introduced by the surface modification on the cathode material; thus, it can be considered that the reaction between the superoxide radical and carbon was inhibited, leading to the suppression of the formation of epoxy groups and carbonates. Furthermore, the enlargement of the discharge product was suppressed during repeated charge–discharge cycles; therefore, the space for generating the discharge product was maintained, and the diffusion path of oxygen and lithium ions required for the discharging reaction was maintained. It is presumed that it could be maintained over a long cycle. Based on this consideration, this phenomenon plays a significant role in improving the cycling performance of KB + VUV.

## 4. Conclusions

This study clarified that the size and shape of the generated discharge product could be changed, and the cycling performance of the Li-air battery could be improved by the surface modification of KB through VUV irradiation, leading to the introduction of the COOH group. This result revealed that the functional group on the carbon surface was one of the important factors that improved the cycling performance. In the future, the cycling performance of Li-air batteries can be further improved by clarifying how the type of the functional group affects the shape of the generated discharge product and the charging–discharging reaction.

## Data Availability

Not applicable.
